# 2-(6-Phenyl-7*H*-1,2,4-triazolo[3,4-*b*][1,3,4]thia­diazin-3-yl)-1,3-benzothia­zole

**DOI:** 10.1107/S1600536811036452

**Published:** 2011-09-14

**Authors:** Hatem A. Abdel-Aziz, Seik Weng Ng, Edward R. T. Tiekink

**Affiliations:** a Department of Pharmaceutical Chemistry, College of Pharmacy, King Saud University, Riyadh 11451, Saudi Arabia; b Department of Chemistry, University of Malaya, 50603 Kuala Lumpur, Malaysia; c Chemistry Department, Faculty of, Science, King Abdulaziz University, PO Box 80203 Jeddah, Saudi Arabia

## Abstract

In the title compound, C_17_H_11_N_5_S_2_, the dihedral angles formed between the triazole ring and the benzene ring and the 1,3-benzothia­zole ring system are 8.67 (8) and 13.90 (9)°, respectively. The conformation of the triazolo-thia­diazin-3-yl fused ring system is a twisted half-chair. Overall, the mol­ecule adopts a flattened shape. Supra­molecular helical chains along the *a* axis sustained by C—H⋯N inter­actions are found in the crystal structure. These are linked *via* C—H⋯π contacts as well as π–π [centroid–centroid distance = 3.5911 (12) Å] inter­actions between the triazole and thia­zole rings.

## Related literature

For background to the synthesis and biological activity of benzothia­zoles and [1,2,4]triazolo[3,4-*b*][1,3,4]thia­diazines, see: Abdel-Aziz *et al.* (2007 ▶, 2010 ▶); Dawood *et al.* (2005 ▶).
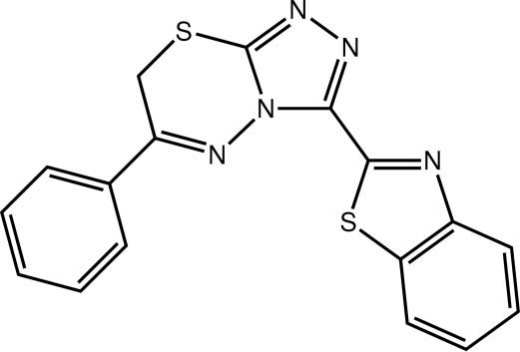


## Experimental

### 

#### Crystal data

C_17_H_11_N_5_S_2_
*M* *_r_* = 349.43
Orthorhombic, 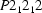

*a* = 12.1437 (3) Å
*b* = 21.2950 (5) Å
*c* = 5.7946 (1) Å
*V* = 1498.48 (6) Å^3^
*Z* = 4
Cu *K*α radiation
μ = 3.29 mm^−1^
*T* = 100 K
0.25 × 0.25 × 0.05 mm

#### Data collection

Agilent SuperNova Dual diffractometer with Atlas detector
Absorption correction: multi-scan (*CrysAlis PRO*; Agilent, 2010 ▶) *T*_min_ = 0.715, *T*_max_ = 1.000
5754 measured reflections
2902 independent reflections
2751 reflections with *I* > 2σ(*I*)
*R*_int_ = 0.029

#### Refinement

*R*[*F* ^2^ > 2σ(*F* ^2^)] = 0.032
*wR*(*F* ^2^) = 0.083
*S* = 1.06
2902 reflections
217 parameters
H-atom parameters constrained
Δρ_max_ = 0.21 e Å^−3^
Δρ_min_ = −0.30 e Å^−3^
Absolute structure: Flack (1983 ▶), 1150 Friedel pairs
Flack parameter: −0.006 (16)

### 

Data collection: *CrysAlis PRO* (Agilent, 2010 ▶); cell refinement: *CrysAlis PRO*; data reduction: *CrysAlis PRO*; program(s) used to solve structure: *SHELXS97* (Sheldrick, 2008 ▶); program(s) used to refine structure: *SHELXL97* (Sheldrick, 2008 ▶); molecular graphics: *ORTEP-3* (Farrugia, 1997 ▶) and *DIAMOND* (Brandenburg, 2006 ▶); software used to prepare material for publication: *publCIF* (Westrip, 2010 ▶).

## Supplementary Material

Crystal structure: contains datablock(s) global, I. DOI: 10.1107/S1600536811036452/hg5092sup1.cif

Structure factors: contains datablock(s) I. DOI: 10.1107/S1600536811036452/hg5092Isup2.hkl

Supplementary material file. DOI: 10.1107/S1600536811036452/hg5092Isup3.cml

Additional supplementary materials:  crystallographic information; 3D view; checkCIF report

## Figures and Tables

**Table 1:** Hydrogen-bond geometry (Å, °) *Cg*1 is the centroid of the C12–C17 ring.

*D*—H⋯*A*	*D*—H	H⋯*A*	*D*⋯*A*	*D*—H⋯*A*
C10—H10a⋯N3^i^	0.99	2.45	3.333 (3)	148
C3—H3⋯*Cg*1^ii^	0.95	2.65	3.377 (2)	134

Symmetry codes: (i) 
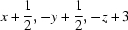
; (ii) 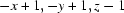.

**Fig. 1.**
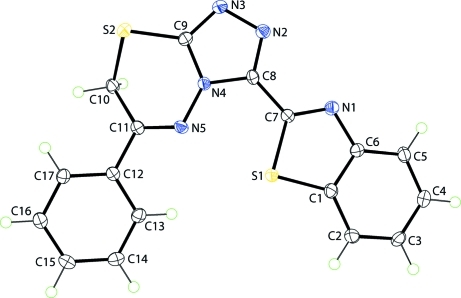
The molecular structure of (I) showing the atom-labelling scheme and displacement ellipsoids at the 50% probability level.

**Fig. 2.**
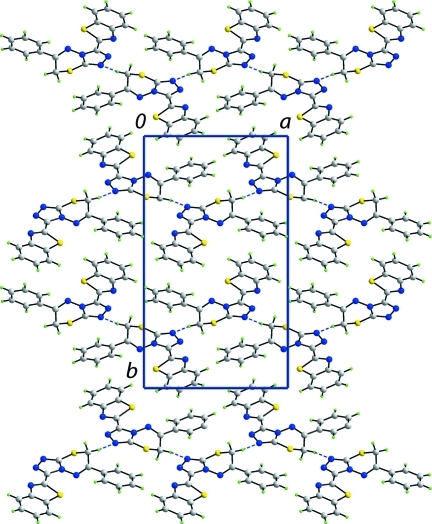
Supramolecular chains in (I) mediated by C—H···N interactions (blue dashed lines).

**Fig. 3.**
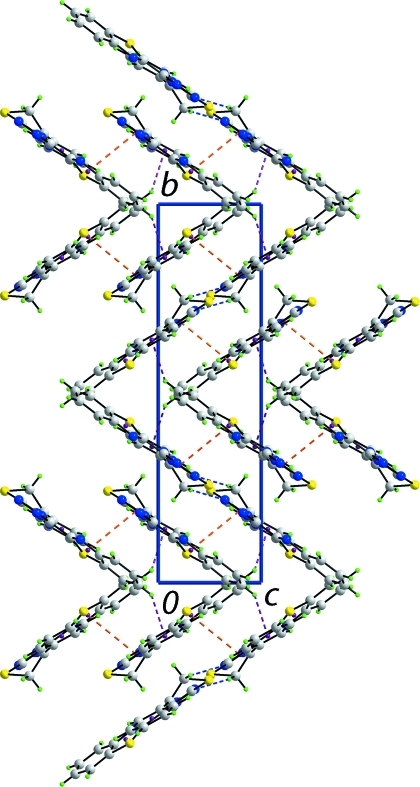
A view in projection down the a axis of the unit-cell contents of (I). The C—H···N, C—H···π and π···π interactions are shown as blue, purple and orange dashed lines, respectively.

## supplementary crystallographic information

### Comment

The title compound, (I), was investigated in relation to the established biological activities exhibited by benzothiazoles and [1,2,4]triazolo[3,4-*b*][1,3,4]thiadiazines (Abdel-Aziz *et al.* 2007; Abdel-Aziz *et al.* 2010; Dawood *et al.* 2005).

In (I), Fig. 1, the 1,3-benzothiazole ring is planar with r.m.s. deviations of 0.034 Å. By contrast, the triazolo-thiadiazin-3-yl fused ring system has a twisted half-chair form owing to the presence of the methylene-C10 group with the C10 atom lying 0.702 (3) Å out of the least-squares plane defined by the S2,N4,N5,C9,C11 atoms (r.m.s. deviation = 0.109 Å). The 1,3-benzothiazole ring forms dihedral angles of 13.90 (9) and 8.67 (8) °, respectively, with the triazole and benzene rings so that the entire molecule has a flattened shape.

In the crystal packing, C—H···N interactions, Table 1, lead to the formation of supramolecular chains along the *a* axis with an helical topology, Fig. 2. These assemble into zigzag layers in the *ac* plane with connections between them of the type C—H···π involving a methylene-H and the benzene ring, and π···π. The shortest interaction of the latter type of 3.5911 (12) Å occurs between the S1,N1,C1,C6,C7 and N2–N4,C8,C9 five-membered rings, Fig. 3.

### Experimental

The title compound was prepared according to the reported method (Abdel-Aziz *et al.*, 2007). Colourless crystals were obtained from an EtOH/DMF (*v*/*v* = 2/1) solution by slow evaporation at room temperature.

### Refinement

Carbon-bound H-atoms were placed in calculated positions [C—H 0.95 to 0.99 Å, *U*_iso_(H) 1.2*U*_eq_(C)] and were included in the refinement in the riding model approximation.

### Crystal data

**Table:**

C_17_H_11_N_5_S_2_	*F*(000) = 720
*M**_r_* = 349.43	*D*_x_ = 1.549 Mg m^−^^3^
Orthorhombic, *P*2_1_2_1_2	Cu *K*α radiation, λ = 1.5418 Å
Hall symbol: P 2 2ab	Cell parameters from 3513 reflections
*a* = 12.1437 (3) Å	θ = 3.6–74.3°
*b* = 21.2950 (5) Å	µ = 3.29 mm^−^^1^
*c* = 5.7946 (1) Å	*T* = 100 K
*V* = 1498.48 (6) Å^3^	Plate, light-brown
*Z* = 4	0.25 × 0.25 × 0.05 mm

### Data collection

**Table:**

Agilent SuperNova Dual diffractometer with Atlas detector	2902 independent reflections
Radiation source: SuperNova (Cu) X-ray Source	2751 reflections with *I* > 2σ(*I*)
mirror	*R*_int_ = 0.029
Detector resolution: 10.4041 pixels mm^-1^	θ_max_ = 74.5°, θ_min_ = 4.2°
ω scan	*h* = −15→13
Absorption correction: multi-scan (*CrysAlis PRO*; Agilent, 2010)	*k* = −13→26
*T*_min_ = 0.715, *T*_max_ = 1.000	*l* = −6→6
5754 measured reflections	

### Refinement

**Table:**

Refinement on *F*^2^	Secondary atom site location: difference Fourier map
Least-squares matrix: full	Hydrogen site location: inferred from neighbouring sites
*R*[*F*^2^ > 2σ(*F*^2^)] = 0.032	H-atom parameters constrained
*wR*(*F*^2^) = 0.083	*w* = 1/[σ^2^(*F*_o_^2^) + (0.0507*P*)^2^ + 0.021*P*] where *P* = (*F*_o_^2^ + 2*F*_c_^2^)/3
*S* = 1.06	(Δ/σ)_max_ = 0.001
2902 reflections	Δρ_max_ = 0.21 e Å^−^^3^
217 parameters	Δρ_min_ = −0.30 e Å^−^^3^
0 restraints	Absolute structure: Flack (1983), 1150 Friedel pairs
Primary atom site location: structure-invariant direct methods	Flack parameter: −0.006 (16)

### Special details

**Table:**

Geometry. All e.s.d.'s (except the e.s.d. in the dihedral angle between two l.s. planes) are estimated using the full covariance matrix. The cell e.s.d.'s are taken into account individually in the estimation of e.s.d.'s in distances, angles and torsion angles; correlations between e.s.d.'s in cell parameters are only used when they are defined by crystal symmetry. An approximate (isotropic) treatment of cell e.s.d.'s is used for estimating e.s.d.'s involving l.s. planes.
Refinement. Refinement of *F*^2^ against ALL reflections. The weighted *R*-factor *wR* and goodness of fit *S* are based on *F*^2^, conventional *R*-factors *R* are based on *F*, with *F* set to zero for negative *F*^2^. The threshold expression of *F*^2^ > σ(*F*^2^) is used only for calculating *R*-factors(gt) *etc*. and is not relevant to the choice of reflections for refinement. *R*-factors based on *F*^2^ are statistically about twice as large as those based on *F*, and *R*- factors based on ALL data will be even larger.

### Fractional atomic coordinates and isotropic or equivalent isotropic displacement parameters (Å^2^)

**Table:**

	*x*	*y*	*z*	*U*_iso_*/*U*_eq_	
S1	0.41692 (4)	0.42415 (2)	0.72377 (10)	0.01863 (14)	
S2	0.51497 (4)	0.25815 (2)	1.48903 (10)	0.01803 (13)	
N1	0.21298 (15)	0.38633 (8)	0.7555 (3)	0.0165 (4)	
N2	0.24914 (15)	0.31575 (9)	1.1796 (3)	0.0170 (4)	
N3	0.30068 (16)	0.28150 (8)	1.3539 (3)	0.0177 (4)	
N4	0.42913 (15)	0.32303 (8)	1.1339 (3)	0.0142 (4)	
N5	0.52940 (15)	0.34569 (8)	1.0531 (3)	0.0155 (4)	
C1	0.32276 (18)	0.45294 (9)	0.5251 (4)	0.0173 (4)	
C2	0.34046 (19)	0.49351 (11)	0.3396 (4)	0.0214 (5)	
H2	0.4105	0.5121	0.3138	0.026*	
C3	0.25298 (18)	0.50565 (10)	0.1955 (4)	0.0204 (5)	
H3	0.2635	0.5325	0.0664	0.024*	
C4	0.14936 (18)	0.47958 (9)	0.2342 (4)	0.0202 (5)	
H4	0.0908	0.4887	0.1309	0.024*	
C5	0.13077 (18)	0.44076 (10)	0.4203 (4)	0.0191 (5)	
H5	0.0597	0.4237	0.4478	0.023*	
C6	0.21838 (18)	0.42704 (9)	0.5676 (4)	0.0149 (4)	
C7	0.30956 (18)	0.38046 (9)	0.8490 (4)	0.0139 (4)	
C8	0.32714 (17)	0.34057 (9)	1.0515 (4)	0.0149 (4)	
C9	0.40792 (19)	0.28660 (9)	1.3227 (4)	0.0153 (4)	
C10	0.61131 (16)	0.25386 (9)	1.2505 (4)	0.0175 (4)	
H10A	0.6859	0.2451	1.3118	0.021*	
H10B	0.5904	0.2184	1.1489	0.021*	
C11	0.61482 (18)	0.31330 (9)	1.1098 (4)	0.0143 (4)	
C12	0.72157 (16)	0.33574 (8)	1.0201 (4)	0.0140 (4)	
C13	0.72667 (19)	0.36733 (9)	0.8082 (4)	0.0171 (4)	
H13	0.6617	0.3728	0.7193	0.021*	
C14	0.82592 (18)	0.39051 (9)	0.7279 (4)	0.0180 (4)	
H14	0.8288	0.4122	0.5846	0.022*	
C15	0.92123 (19)	0.38220 (10)	0.8562 (4)	0.0186 (4)	
H15	0.9891	0.3985	0.8010	0.022*	
C16	0.91816 (19)	0.35013 (10)	1.0651 (4)	0.0184 (4)	
H16	0.9838	0.3443	1.1518	0.022*	
C17	0.81865 (18)	0.32671 (9)	1.1466 (4)	0.0159 (4)	
H17	0.8164	0.3045	1.2886	0.019*	

### Atomic displacement parameters (Å^2^)

**Table:**

	*U*^11^	*U*^22^	*U*^33^	*U*^12^	*U*^13^	*U*^23^
S1	0.0133 (2)	0.0176 (2)	0.0250 (3)	−0.00121 (19)	−0.0008 (2)	0.0079 (2)
S2	0.0186 (3)	0.0185 (3)	0.0170 (3)	0.00037 (19)	−0.0012 (2)	0.00396 (19)
N1	0.0172 (9)	0.0139 (8)	0.0184 (9)	−0.0010 (7)	−0.0006 (8)	0.0007 (8)
N2	0.0168 (9)	0.0155 (8)	0.0186 (9)	−0.0002 (7)	0.0009 (8)	0.0010 (7)
N3	0.0185 (9)	0.0171 (9)	0.0175 (10)	−0.0008 (7)	0.0015 (7)	0.0035 (7)
N4	0.0129 (9)	0.0131 (8)	0.0167 (9)	−0.0003 (7)	−0.0003 (7)	0.0014 (7)
N5	0.0134 (9)	0.0136 (8)	0.0195 (10)	−0.0017 (7)	0.0019 (7)	0.0015 (7)
C1	0.0158 (10)	0.0138 (9)	0.0224 (11)	0.0024 (8)	0.0009 (9)	0.0014 (9)
C2	0.0168 (11)	0.0181 (10)	0.0295 (13)	−0.0004 (9)	0.0018 (10)	0.0066 (9)
C3	0.0231 (12)	0.0158 (10)	0.0223 (11)	0.0039 (9)	0.0017 (10)	0.0065 (9)
C4	0.0201 (11)	0.0195 (10)	0.0211 (12)	0.0042 (8)	−0.0038 (10)	0.0012 (9)
C5	0.0139 (11)	0.0198 (10)	0.0237 (12)	0.0009 (8)	−0.0021 (9)	0.0014 (9)
C6	0.0173 (11)	0.0118 (8)	0.0157 (10)	0.0011 (8)	0.0013 (8)	−0.0008 (8)
C7	0.0137 (9)	0.0105 (9)	0.0175 (11)	−0.0002 (8)	0.0008 (8)	−0.0011 (8)
C8	0.0127 (10)	0.0138 (9)	0.0183 (11)	0.0017 (8)	−0.0010 (8)	−0.0012 (8)
C9	0.0176 (10)	0.0121 (9)	0.0161 (10)	0.0003 (8)	0.0001 (8)	0.0004 (7)
C10	0.0139 (9)	0.0134 (9)	0.0252 (11)	0.0001 (8)	−0.0016 (9)	0.0024 (9)
C11	0.0145 (10)	0.0122 (9)	0.0161 (10)	−0.0022 (8)	−0.0012 (8)	−0.0024 (8)
C12	0.0143 (10)	0.0093 (8)	0.0184 (10)	−0.0001 (7)	−0.0001 (8)	−0.0015 (8)
C13	0.0199 (11)	0.0124 (9)	0.0191 (11)	−0.0002 (8)	−0.0015 (9)	0.0005 (8)
C14	0.0218 (11)	0.0159 (9)	0.0161 (11)	0.0000 (8)	0.0033 (9)	0.0007 (9)
C15	0.0160 (10)	0.0180 (10)	0.0219 (11)	−0.0031 (9)	0.0039 (9)	−0.0027 (9)
C16	0.0161 (10)	0.0169 (9)	0.0221 (12)	0.0003 (8)	−0.0015 (9)	−0.0010 (8)
C17	0.0175 (11)	0.0123 (9)	0.0177 (11)	0.0013 (8)	−0.0017 (9)	−0.0002 (8)

### Geometric parameters (Å, °)

**Table:**

S1—C1	1.734 (2)	C4—H4	0.9500
S1—C7	1.758 (2)	C5—C6	1.395 (3)
S2—C9	1.728 (2)	C5—H5	0.9500
S2—C10	1.813 (2)	C7—C8	1.464 (3)
N1—C7	1.298 (3)	C10—C11	1.507 (3)
N1—C6	1.393 (3)	C10—H10A	0.9900
N2—C8	1.314 (3)	C10—H10B	0.9900
N2—N3	1.394 (3)	C11—C12	1.476 (3)
N3—C9	1.319 (3)	C12—C13	1.402 (3)
N4—C9	1.366 (3)	C12—C17	1.401 (3)
N4—C8	1.379 (3)	C13—C14	1.383 (3)
N4—N5	1.391 (2)	C13—H13	0.9500
N5—C11	1.288 (3)	C14—C15	1.387 (3)
C1—C2	1.396 (3)	C14—H14	0.9500
C1—C6	1.404 (3)	C15—C16	1.391 (3)
C2—C3	1.376 (3)	C15—H15	0.9500
C2—H2	0.9500	C16—C17	1.390 (3)
C3—C4	1.394 (3)	C16—H16	0.9500
C3—H3	0.9500	C17—H17	0.9500
C4—C5	1.377 (3)		
			
C1—S1—C7	88.40 (10)	N4—C8—C7	124.43 (19)
C9—S2—C10	94.47 (10)	N3—C9—N4	110.04 (19)
C7—N1—C6	110.09 (18)	N3—C9—S2	129.60 (17)
C8—N2—N3	107.21 (18)	N4—C9—S2	120.26 (17)
C9—N3—N2	107.51 (17)	C11—C10—S2	112.86 (14)
C9—N4—C8	105.17 (18)	C11—C10—H10A	109.0
C9—N4—N5	129.18 (19)	S2—C10—H10A	109.0
C8—N4—N5	125.17 (17)	C11—C10—H10B	109.0
C11—N5—N4	115.69 (17)	S2—C10—H10B	109.0
C2—C1—C6	121.1 (2)	H10A—C10—H10B	107.8
C2—C1—S1	128.93 (17)	N5—C11—C12	116.37 (18)
C6—C1—S1	109.87 (16)	N5—C11—C10	124.40 (19)
C3—C2—C1	117.7 (2)	C12—C11—C10	119.17 (18)
C3—C2—H2	121.1	C13—C12—C17	119.1 (2)
C1—C2—H2	121.1	C13—C12—C11	120.2 (2)
C2—C3—C4	121.6 (2)	C17—C12—C11	120.7 (2)
C2—C3—H3	119.2	C14—C13—C12	120.3 (2)
C4—C3—H3	119.2	C14—C13—H13	119.8
C5—C4—C3	120.9 (2)	C12—C13—H13	119.8
C5—C4—H4	119.6	C13—C14—C15	120.1 (2)
C3—C4—H4	119.6	C13—C14—H14	120.0
C4—C5—C6	118.7 (2)	C15—C14—H14	120.0
C4—C5—H5	120.7	C14—C15—C16	120.5 (2)
C6—C5—H5	120.7	C14—C15—H15	119.8
C5—C6—N1	124.9 (2)	C16—C15—H15	119.8
C5—C6—C1	119.93 (19)	C17—C16—C15	119.7 (2)
N1—C6—C1	115.08 (19)	C17—C16—H16	120.2
N1—C7—C8	121.47 (19)	C15—C16—H16	120.2
N1—C7—S1	116.54 (16)	C16—C17—C12	120.3 (2)
C8—C7—S1	121.97 (16)	C16—C17—H17	119.8
N2—C8—N4	110.06 (18)	C12—C17—H17	119.8
N2—C8—C7	125.50 (19)		
			
C8—N2—N3—C9	−0.5 (2)	S1—C7—C8—N2	166.92 (17)
C9—N4—N5—C11	25.6 (3)	N1—C7—C8—N4	167.6 (2)
C8—N4—N5—C11	−163.6 (2)	S1—C7—C8—N4	−13.8 (3)
C7—S1—C1—C2	−177.9 (2)	N2—N3—C9—N4	0.0 (2)
C7—S1—C1—C6	−0.48 (16)	N2—N3—C9—S2	176.46 (16)
C6—C1—C2—C3	−2.0 (3)	C8—N4—C9—N3	0.5 (2)
S1—C1—C2—C3	175.07 (18)	N5—N4—C9—N3	172.72 (19)
C1—C2—C3—C4	1.2 (4)	C8—N4—C9—S2	−176.35 (15)
C2—C3—C4—C5	0.4 (4)	N5—N4—C9—S2	−4.1 (3)
C3—C4—C5—C6	−1.2 (3)	C10—S2—C9—N3	153.5 (2)
C4—C5—C6—N1	−176.8 (2)	C10—S2—C9—N4	−30.35 (18)
C4—C5—C6—C1	0.3 (3)	C9—S2—C10—C11	48.76 (17)
C7—N1—C6—C5	176.0 (2)	N4—N5—C11—C12	178.39 (18)
C7—N1—C6—C1	−1.3 (3)	N4—N5—C11—C10	1.0 (3)
C2—C1—C6—C5	1.3 (3)	S2—C10—C11—N5	−41.2 (3)
S1—C1—C6—C5	−176.30 (16)	S2—C10—C11—C12	141.45 (17)
C2—C1—C6—N1	178.7 (2)	N5—C11—C12—C13	−30.4 (3)
S1—C1—C6—N1	1.1 (2)	C10—C11—C12—C13	147.11 (19)
C6—N1—C7—C8	179.56 (17)	N5—C11—C12—C17	148.6 (2)
C6—N1—C7—S1	0.9 (2)	C10—C11—C12—C17	−33.9 (3)
C1—S1—C7—N1	−0.26 (18)	C17—C12—C13—C14	−1.5 (3)
C1—S1—C7—C8	−178.90 (18)	C11—C12—C13—C14	177.53 (19)
N3—N2—C8—N4	0.9 (2)	C12—C13—C14—C15	0.5 (3)
N3—N2—C8—C7	−179.81 (18)	C13—C14—C15—C16	0.6 (3)
C9—N4—C8—N2	−0.8 (2)	C14—C15—C16—C17	−0.5 (3)
N5—N4—C8—N2	−173.48 (19)	C15—C16—C17—C12	−0.5 (3)
C9—N4—C8—C7	179.81 (18)	C13—C12—C17—C16	1.5 (3)
N5—N4—C8—C7	7.2 (3)	C11—C12—C17—C16	−177.49 (19)
N1—C7—C8—N2	−11.7 (3)		

### Hydrogen-bond geometry (Å, °)

**Table:**

Cg1 is the centroid of the C12–C17 ring.

**Table:**

*D*—H···*A*	*D*—H	H···*A*	*D*···*A*	*D*—H···*A*
C10—H10a···N3^i^	0.99	2.45	3.333 (3)	148
C3—H3···Cg1^ii^	0.95	2.65	3.377 (2)	134

Symmetry codes: (i) *x*+1/2, −*y*+1/2, −*z*+3; (ii) −*x*+1, −*y*+1, *z*−1.
